# Supplementary material for: Combination of Immune-Related Network and Molecular Typing Analysis Defines a Three-Gene Signature for Predicting Prognosis of Triple-Negative Breast Cancer
Source: Biomolecules. 2022 Oct 25;12(11):1556. doi: 10.3390/biom12111556 (PMC9687467; doi:10.3390/biom12111556)
Supplement: Supplementary file 1 [file biomolecules-12-01556-s001.zip › Supplementary Table. S3.pdf]

Table S3. The results of Cox regression analysis for DEGs between different IRGs subtypes.

id	HR	HR.95L	HR.95H	*p* Value
GBP4	0.826445146	0.739094681	0.924119191	0.000824152
GBP5	0.83977668	0.757725948	0.930712318	0.000872225
CCL5	0.855939412	0.775251657	0.945025104	0.002075143
IRF1	0.798617935	0.694273376	0.918644771	0.001645155
CD2	0.846573567	0.757063487	0.946666715	0.003486463
STAT1	0.869857153	0.769429363	0.983393023	0.025913278
ICOS	0.758565913	0.640874867	0.897869888	0.001316524
APOBEC3G	0.799921208	0.686160572	0.932542563	0.004340492
CD3D	0.836624816	0.748604511	0.934994475	0.001660669
IDO1	0.845236506	0.768624095	0.929485239	0.000523611
TAP1	0.837694746	0.743861071	0.943364985	0.00347975
CXCR3	0.811014689	0.692347456	0.950021294	0.009454309
GZMB	0.842571049	0.759994762	0.93411956	0.001134124
NKG7	0.895671053	0.803607464	0.998281713	0.046476386
CTLA4	0.733047684	0.615367154	0.873233003	0.000504792
PSMB9	0.8491472	0.747369538	0.964785065	0.012062424
HLA-F	0.800900787	0.698084173	0.918860641	0.001539812
CXCL9	0.877542166	0.814707322	0.945223189	0.00056877
CD8A	0.830216289	0.733185987	0.940087643	0.003343558
CD247	0.809391756	0.708594677	0.924527145	0.001830797
CD38	0.828476693	0.725892495	0.945558241	0.005270977
CXCR6	0.723309482	0.594215414	0.880449404	0.001240878
PSMB10	0.769976227	0.637455846	0.930046204	0.006677393
GBP1	0.816051092	0.723157216	0.920877743	0.000977964
AIM2	0.837357619	0.728101372	0.96300846	0.01283298
IL18BP	0.832042422	0.706388364	0.980048125	0.02771945
GZMA	0.844759191	0.750531825	0.950816563	0.005177804
UBD	0.880788466	0.818400045	0.947932893	0.000707898
HLA-B	0.820318009	0.714936483	0.941232754	0.004753469
CYTIP	0.804093142	0.667399641	0.96878353	0.021815529
CD7	0.821873132	0.708017447	0.954037853	0.009925792
HLA-E	0.830964711	0.696811107	0.990946247	0.039283464
CXCL13	0.849843133	0.774866107	0.932075031	0.000555088
CD48	0.842756097	0.739077671	0.960978618	0.010642138
IL18RAP	0.605363605	0.464897734	0.78827034	0.0001944
CD3G	0.790930055	0.659502203	0.948549298	0.011417185
PRF1	0.824478436	0.70625494	0.962491946	0.014522288
ITK	0.800498826	0.678253309	0.944777359	0.008492648
LTA	0.801350358	0.659829147	0.973225264	0.025502412
CORO1A	0.798557407	0.684887553	0.931092892	0.004088177
CXCL10	0.917181314	0.847027544	0.993145463	0.033222692
SASH3	0.836190153	0.71185204	0.982246214	0.029402043
GIMAP4	0.84468308	0.726255639	0.982421984	0.028519645

ZBED2	0.71634754	0.575698528	0.891358536	0.002778681
IFNG	0.780000579	0.648846677	0.937665129	0.008164624
CST7	0.835715047	0.715456924	0.976186848	0.023573852
RAC2	0.847508662	0.74357375	0.965971339	0.013189524
MAP4K1	0.855951281	0.734071586	0.998066959	0.047183125
HCST	0.82911561	0.722489254	0.951478089	0.007627168
PDCD1	0.805726218	0.661078838	0.982023173	0.032383126
GNLY	0.837996578	0.75060695	0.935560567	0.001658716
ARHGAP9	0.821661222	0.700640704	0.963585415	0.015680808
SLAMF1	0.776381632	0.636059302	0.947660755	0.01282829
IL2RG	0.789870285	0.656854676	0.949822068	0.012171691
CD6	0.811580161	0.698868826	0.94246922	0.006206621
PYHIN1	0.774349442	0.635905206	0.942934657	0.010939313
CD53	0.836257445	0.709085208	0.986237629	0.033618692
PLEK	0.84178936	0.737180892	0.961242126	0.010964837
SH2D1A	0.758776042	0.634958029	0.906738801	0.002388984
LAMP3	0.839905076	0.748042642	0.94304856	0.00315538
CD27	0.825652793	0.713909073	0.954887057	0.009818931
HLA-DMA	0.830247696	0.719828873	0.957604318	0.01062115
HLA-DMB	0.830632207	0.722643368	0.954758451	0.009014597
TBC1D10C	0.810956743	0.697181094	0.943299876	0.006592353
RASAL3	0.839282458	0.719467946	0.979049934	0.02578949
PSTPIP1	0.80790319	0.653080298	0.999429268	0.049388368
ARHGAP25	0.828309731	0.69375355	0.988963602	0.037281803
ZNF683	0.815035462	0.695944188	0.954505858	0.011158675
NLRC5	0.745806182	0.613910377	0.906039191	0.003140035
BTN3A1	0.755750754	0.621730266	0.918660765	0.00492673
ACAP1	0.817536862	0.687299651	0.972452872	0.022877543
GZMK	0.870754081	0.784646488	0.966311174	0.009187037
PRKCB	0.814846315	0.707605194	0.938340367	0.004456304
HCLS1	0.817673893	0.706015876	0.94699088	0.007209204
STAT4	0.748664266	0.623681301	0.898693264	0.00189546
PSME2	0.7409021	0.605088627	0.907199205	0.003700236
HLA-DOB	0.784192549	0.673837099	0.912621099	0.001680499
SEMA4D	0.766928207	0.624937438	0.941180412	0.011076529
IL10RA	0.831649855	0.706978893	0.978305701	0.026104657
TNFRSF1B	0.781459707	0.64229019	0.95078406	0.013727338
SLAMF7	0.801879498	0.668832621	0.961392596	0.017064647
BTN3A3	0.733500494	0.602857554	0.892454564	0.001955656
FGL2	0.865348898	0.759523293	0.985919356	0.029777493
CTSW	0.818501692	0.691340944	0.969051559	0.020077424
GPSM3	0.798230112	0.664460035	0.958930978	0.01603691
GZMH	0.83360377	0.71663248	0.969667528	0.018311661
CYBB	0.868197321	0.760186817	0.991554407	0.037061294
SLA	0.799452427	0.652080354	0.980131021	0.031318027
ZAP70	0.821307826	0.683489016	0.986916438	0.035685911
ARHGAP30	0.772577925	0.639148636	0.933862043	0.007645664
PIM2	0.838810551	0.732245188	0.960884622	0.011227128
IL7R	0.855881478	0.761346398	0.962154819	0.009160433
CCR7	0.860718451	0.756130754	0.979772676	0.023261433
AKNA	0.815411783	0.678064825	0.980579365	0.030131514

FASLG	0.78741369	0.62479804	0.992353177	0.042867089
GIMAP5	0.831330044	0.69350162	0.996550868	0.045790305
P2RY8	0.827227127	0.700020659	0.977549322	0.025980464
S1PR4	0.790165307	0.638107434	0.978457826	0.030798576
EVI2B	0.860907208	0.749961322	0.988265927	0.033366389
SAMD3	0.720804952	0.555714211	0.934940601	0.013630323
UBASH3A	0.7361444	0.569106384	0.952209628	0.019653156
RTP4	0.839356696	0.730480911	0.964460059	0.013493841
IRF8	0.83070884	0.72947177	0.945995727	0.005154066
GIMAP7	0.866232807	0.753479159	0.995859364	0.043562079
TNFAIP3	0.821776344	0.686408918	0.983839721	0.032567849
PATL2	0.719761764	0.538073696	0.962799335	0.026734956
TNFSF13B	0.848778092	0.747910231	0.963249624	0.011084454
STX11	0.78590372	0.651448613	0.948109559	0.011850712
HLA-DRA	0.841891908	0.749449318	0.945737046	0.003730661
SELL	0.846843909	0.746196948	0.961066121	0.01002042
GBP2	0.819034855	0.71239071	0.941643517	0.005035344
HLA-DQA1	0.869346296	0.784589315	0.963259337	0.007469156
APOBEC3F	0.779505145	0.637254199	0.953510031	0.01539139
CD69	0.815312609	0.700156192	0.949409086	0.008583564
P2RY10	0.804765295	0.669616057	0.967191832	0.02058042
LTB	0.853589388	0.769462907	0.946913538	0.002786507
LY9	0.774045948	0.607904297	0.985594496	0.037739683
GMFG	0.804452821	0.676503574	0.956601511	0.013817485
LCP2	0.743774794	0.579986189	0.953817445	0.01967217
HLA-DOA	0.843121997	0.739621905	0.961105529	0.010660669
PSMB8	0.841086459	0.72320464	0.978182925	0.024686216
TRAT1	0.735227336	0.589276173	0.917327494	0.006444606
HLA-DPA1	0.840036578	0.747207067	0.944398793	0.003529197
LAT2	0.791764521	0.637700688	0.983049054	0.034446086
PSME1	0.774230118	0.627040744	0.955970216	0.017382617
DOCK10	0.800382482	0.668643554	0.958077161	0.015236348
TRAF1	0.735484633	0.577188509	0.937194067	0.012972457
SAMSN1	0.825311759	0.683416018	0.996668913	0.046083418
ARHGAP15	0.82160851	0.689413323	0.979152159	0.028138161
BTLA	0.728873738	0.565524823	0.93940514	0.014574382
PTPRC	0.816449706	0.688310886	0.968443382	0.019907778
LPXN	0.80864644	0.677407449	0.965311299	0.018737898
IGSF6	0.844156167	0.714846037	0.9968575	0.045818066
HLA-G	0.820699656	0.705118216	0.955226954	0.010728375
IL15RA	0.693617039	0.551946866	0.871650199	0.001698745
DOCK2	0.849392021	0.728321339	0.990588586	0.037481163
IL32	0.855495229	0.747979166	0.978465873	0.022747334
TNFRSF14	0.76753386	0.635761001	0.926619006	0.005905366
CD79A	0.876819034	0.802026757	0.958585996	0.00385546
NFS1	0.778063878	0.654265563	0.925286967	0.0045369
BTN3A2	0.79909998	0.675495468	0.945322076	0.00890163
CD52	0.859034743	0.776242678	0.950657198	0.003297135
TAPBP	0.816015671	0.667910839	0.996961774	0.046619379
IRF9	0.76398186	0.61965468	0.941925079	0.011735092
CD40	0.778917407	0.641747482	0.945406636	0.011471717

PTPN6	0.80792156	0.657072201	0.993402623	0.043100221
HLA-H	0.833232559	0.740123048	0.938055501	0.002547411
RASSF5	0.760106107	0.641932142	0.900034843	0.001464626
TRAF3IP3	0.778557961	0.651738219	0.930055167	0.005792795
P2RY13	0.775815228	0.613103023	0.981709835	0.034543312
THEMIS	0.679895945	0.514293068	0.898823114	0.006750334
IGFLR1	0.80800525	0.66088791	0.987871732	0.037621044
ZNF831	0.747132144	0.571651229	0.976480785	0.03282571
PARVG	0.808050242	0.664227395	0.98301455	0.033066519
PLAC8	0.835978193	0.72105083	0.969223681	0.017585208
LCP1	0.812300127	0.713122826	0.925270477	0.001753961
TRIM69	0.636325228	0.478716669	0.845823474	0.001851292
TLR8	0.835249563	0.697663974	0.999968263	0.049959617
IL23A	0.727688299	0.560380407	0.944947851	0.017089715
CXCL11	0.842307772	0.751576652	0.943992048	0.003165951
CD79B	0.841919307	0.74000919	0.957863942	0.008950775
DEF6	0.688674499	0.54123386	0.876280291	0.002410119
TNFRSF17	0.858923358	0.759410533	0.9714763	0.01549602
SPOCK2	0.834789829	0.739460071	0.942409315	0.00351491
HLA-DPB1	0.842457808	0.721242413	0.984045233	0.030550282
IGHG1	0.911837926	0.858585215	0.968393573	0.002646952
GIMAP6	0.833482179	0.705265175	0.985008997	0.032582189
SRGN	0.855908187	0.758890246	0.965329082	0.011250213
CLECL1	0.705965455	0.560294059	0.889510099	0.003147633
PIK3CD	0.795769389	0.64999929	0.974230172	0.026907431
KLRB1	0.791043025	0.677702991	0.923338213	0.002969804
NAPSB	0.844419394	0.73958465	0.964114266	0.012408673
TNFRSF4	0.802306827	0.646491511	0.995676253	0.045576457
CD19	0.850620577	0.747224222	0.968324293	0.014415113
ACSL5	0.837514276	0.715091546	0.980895616	0.027863582
PTPN22	0.778305876	0.612391389	0.989171382	0.040464871
MZB1	0.893043964	0.817034764	0.976124341	0.012688016
ALOX5	0.834113909	0.712620334	0.976320742	0.023925222
CCR2	0.774473999	0.623663155	0.961753105	0.020728884
IGKC	0.88120372	0.811261619	0.957175809	0.002724018
TAPBPL	0.651971258	0.518286872	0.820137542	0.000258648
SEL1L3	0.84037614	0.71744197	0.984375164	0.0311522
PRDM1	0.817582788	0.674164046	0.991511812	0.040698745
VCAM1	0.846407804	0.748889504	0.956624665	0.007585572
IL15	0.789442395	0.62845244	0.991672966	0.042171408
KLRD1	0.6380905	0.484437866	0.840478243	0.001391823
CTSC	0.825575808	0.710131369	0.959787788	0.012631135
WIPF1	0.787109834	0.651288649	0.951255472	0.013247186
B2M	0.822561987	0.709391966	0.953786137	0.009695587
PLCB2	0.774263722	0.618789665	0.968801429	0.025278926
SKAP1	0.850040808	0.729652921	0.990291896	0.037054103
IFI16	0.76966276	0.654167708	0.905548772	0.001599684
PAG1	0.68644641	0.549208352	0.857977982	0.000946572
IGLL1	0.84866951	0.755778342	0.952977741	0.005532069
PVRIG	0.787699318	0.645026007	0.961930539	0.019249878
GIMAP2	0.803048766	0.64703924	0.996674206	0.046569896

CSF2RA	0.810523945	0.674247769	0.974343699	0.025306208
REC8	0.777192162	0.625592286	0.965529259	0.022797216
TMEM176B	0.695342797	0.573530216	0.843027258	0.000217573
RAB8B	0.771726244	0.623131069	0.955756222	0.017564947
CD274	0.724188023	0.553246577	0.947946747	0.018818872
FGD3	0.732620753	0.608320677	0.882319454	0.001038851
FAM30A	0.759374858	0.600644362	0.960052589	0.021408057
PPP1R16B	0.794413037	0.676703071	0.932598211	0.004911432
NMI	0.806385451	0.659456053	0.986051297	0.036008056
STAMBPL1	0.679834711	0.547896622	0.843544595	0.000455722
LRMP	0.829837991	0.691888406	0.995292138	0.044342665
IL18R1	0.633562674	0.492244454	0.815451874	0.000393671
GPR18	0.802836573	0.652088367	0.988434383	0.038490189
PLCL2	0.721513233	0.594972825	0.874966592	0.000908082
LYSMD2	0.746713312	0.614727879	0.907036738	0.003248534
PLCG2	0.835597836	0.716123025	0.975005298	0.022518956
MICA	0.791074101	0.644375734	0.971169769	0.025123012
CCL19	0.922260916	0.855984328	0.993669124	0.033430293
LGALS2	0.743259459	0.595175976	0.928187035	0.008861876
CXCR5	0.811547156	0.659873859	0.998082856	0.047912569
RCSD1	0.768285658	0.61589446	0.958383117	0.019449508
RFTN1	0.770885634	0.641816268	0.925910872	0.00538033
FCRL5	0.777896753	0.609378018	0.993018028	0.043778165
LGALS3BP	0.838182975	0.73847937	0.951347767	0.006297948
BIRC3	0.823033845	0.687038796	0.985948265	0.034554864
SNX10	0.788161555	0.660080014	0.941095963	0.008515261
CLEC4A	0.796832566	0.647490479	0.980620037	0.031970397
EFHD2	0.743521135	0.594019116	0.930649643	0.009668213
ANKRD22	0.83069572	0.711096363	0.970410503	0.019353287
C1S	0.836162691	0.734207649	0.952275621	0.00699576
RUNX3	0.794691182	0.690572926	0.914507433	0.001340023
TMEM176A	0.721313182	0.610431449	0.852336011	0.00012497
SUSD3	0.800973566	0.681356281	0.941590578	0.007160841
CELF2	0.761358076	0.614908101	0.942687403	0.012368596
STK17B	0.778375779	0.626973433	0.966338957	0.023194132
PTGER4	0.740359491	0.608102898	0.901380634	0.002753061
MAL	0.863541032	0.749885133	0.994423122	0.041586426
IL2RA	0.783320714	0.638061214	0.961649647	0.01961442
CCND2	0.743063824	0.631381972	0.874500494	0.000351929
GLRX	0.798263487	0.65996802	0.965538594	0.020273164
HSD11B1	0.793451203	0.649652927	0.969078695	0.023338828
RASGRP1	0.732602849	0.59651979	0.899730309	0.002999933
BANK1	0.806807707	0.681213426	0.955557615	0.012899502
PTGDS	0.871291745	0.788453763	0.962833002	0.006871135
TOX2	0.819974213	0.689930538	0.97452957	0.02427162
MARCO	0.900863053	0.814691584	0.996149042	0.041832946
CCL13	0.863806878	0.760913238	0.980614195	0.023667508
ETS1	0.827678754	0.706362221	0.9698312	0.019347189
CD83	0.737648004	0.609626288	0.892554321	0.001755821
BCL11B	0.775193972	0.654317653	0.918400555	0.003238688
TNF	0.839378362	0.714700879	0.985805468	0.032824682

PRKCQ-AS1	0.828216022	0.70504182	0.97290935	0.021774905
RASSF2	0.771163734	0.650045512	0.914849027	0.002874358
ITM2A	0.847534303	0.752297189	0.954827967	0.006527965
TUBB2A	1.167679675	1.001774774	1.36106025	0.047407354
NFE2L3	0.826064853	0.719852069	0.947949129	0.006504293
HLA-A	0.907412868	0.846909591	0.972238503	0.005786365
NDUFA4L2	1.113153408	1.004759429	1.233240987	0.040286291
TMPRSS3	0.871414916	0.768387586	0.988256408	0.032034736
FBP1	0.886667474	0.789416142	0.995899586	0.042429516
GPRC5C	1.211603826	1.081258813	1.357661841	0.000948773
MMP7	0.927306218	0.873633158	0.984276769	0.013103947
AZGP1	1.095925096	1.018233357	1.179544755	0.01462219
REEP6	1.11355715	1.027825421	1.206439831	0.008503297
TFAP2B	1.081932647	1.001571441	1.168741643	0.045517584
SPDEF	1.095665501	1.022909439	1.173596455	0.009158587
SPINK8	1.10264145	1.023208864	1.188240457	0.010424185
GABRP	0.934842525	0.887035427	0.985226204	0.011879761
FOXA1	1.070564683	1.002607458	1.143128082	0.041571133
TFF3	1.06715226	1.006335079	1.131644885	0.02993881

HR:Hazard ratios; HR.95L: 95% lower confidence intervals; HR.95H: 95% higher confidence intervals;
