# Supplementary figures and images for: Combination of Immune-Related Network and Molecular Typing Analysis Defines a Three-Gene Signature for Predicting Prognosis of Triple-Negative Breast Cancer
Source: Biomolecules. 2022 Oct 25;12(11):1556. doi: 10.3390/biom12111556 (PMC9687467; doi:10.3390/biom12111556)

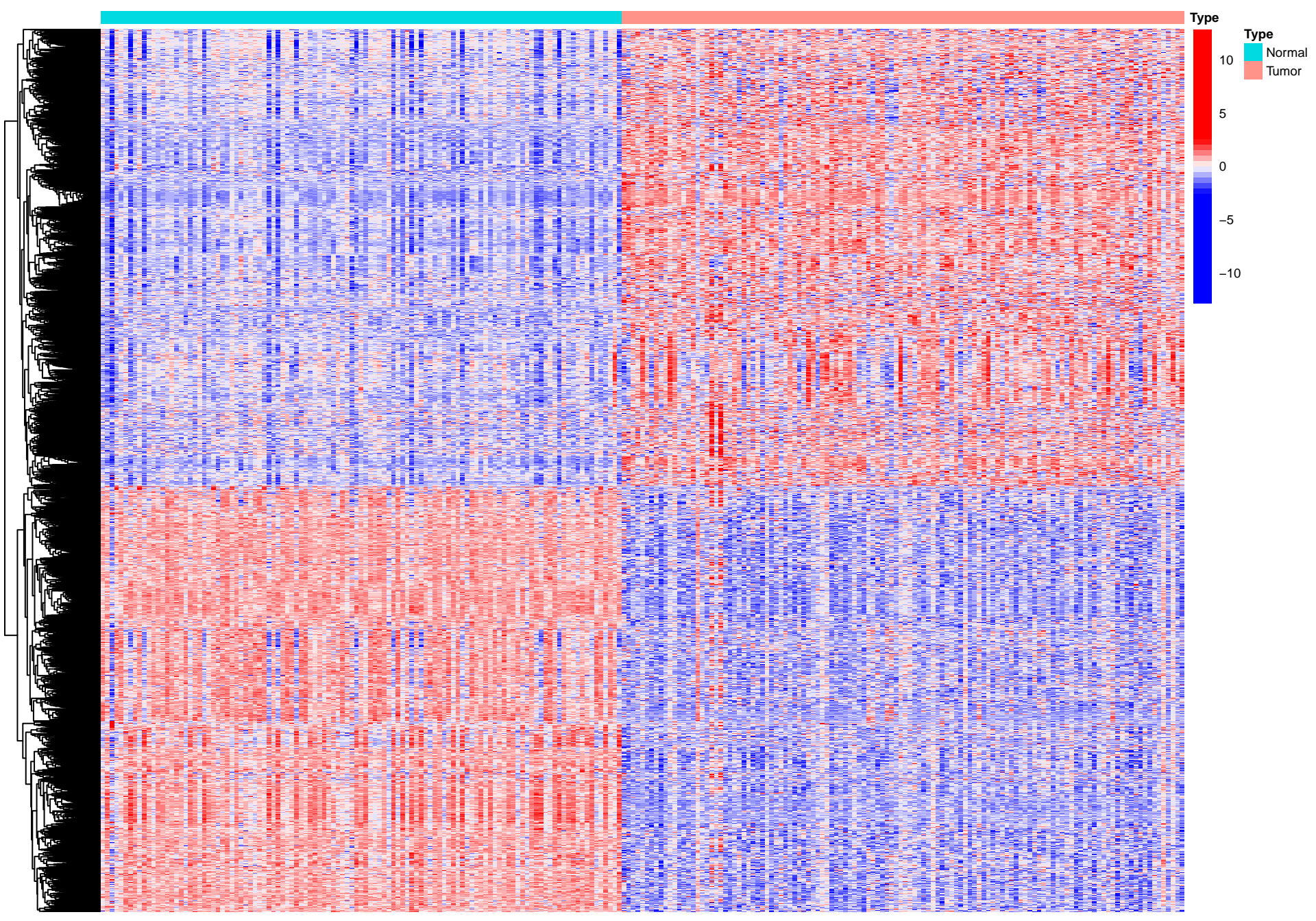

Supplement: Supplementary file 1 [file biomolecules-12-01556-s001.zip › Supplementary Figure S1.pdf]

## Sample clustering to detect outliers

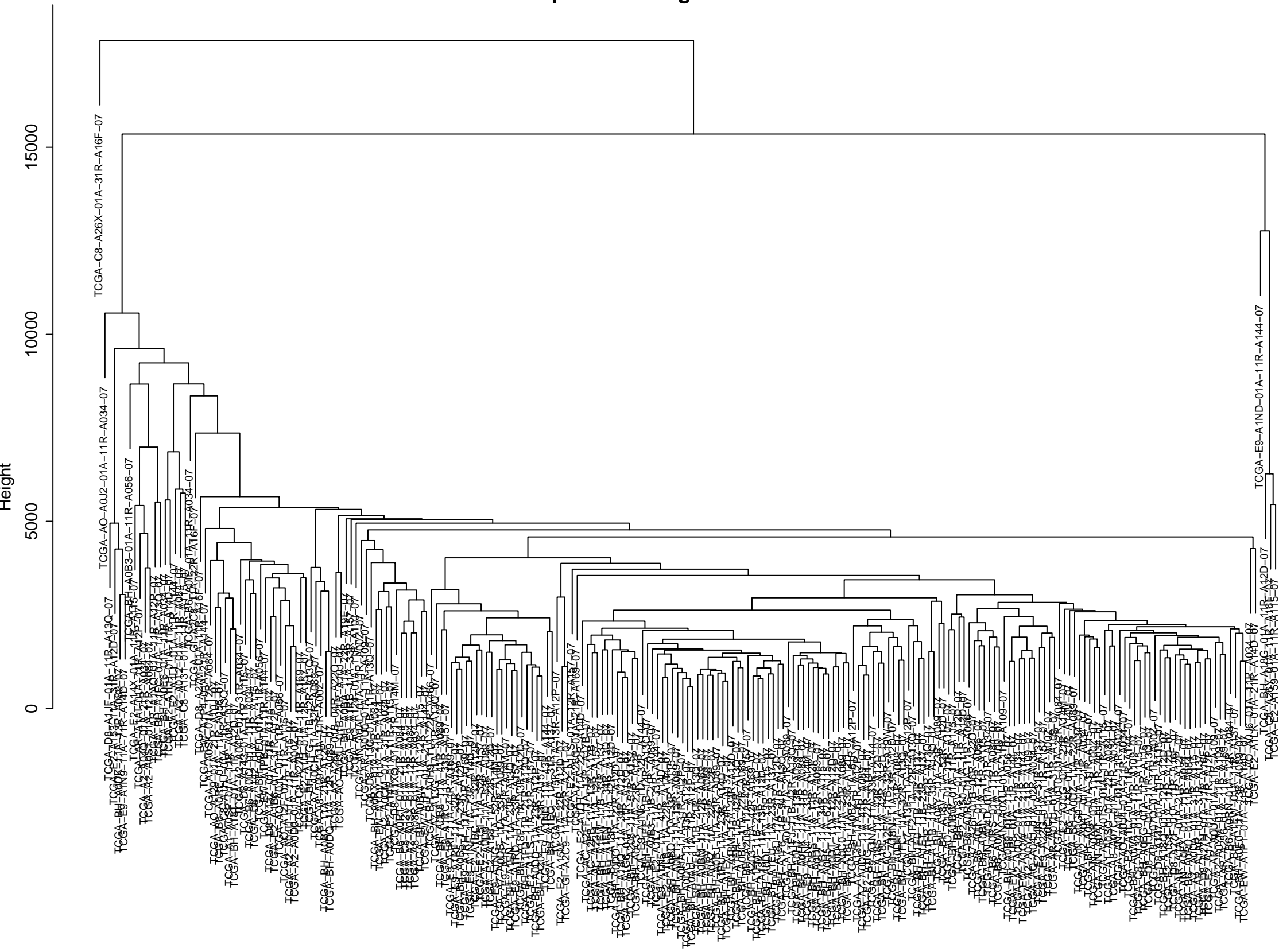

Supplement: Supplementary file 1 [file biomolecules-12-01556-s001.zip › Supplementary Figure S2.pdf]

### Scale independence

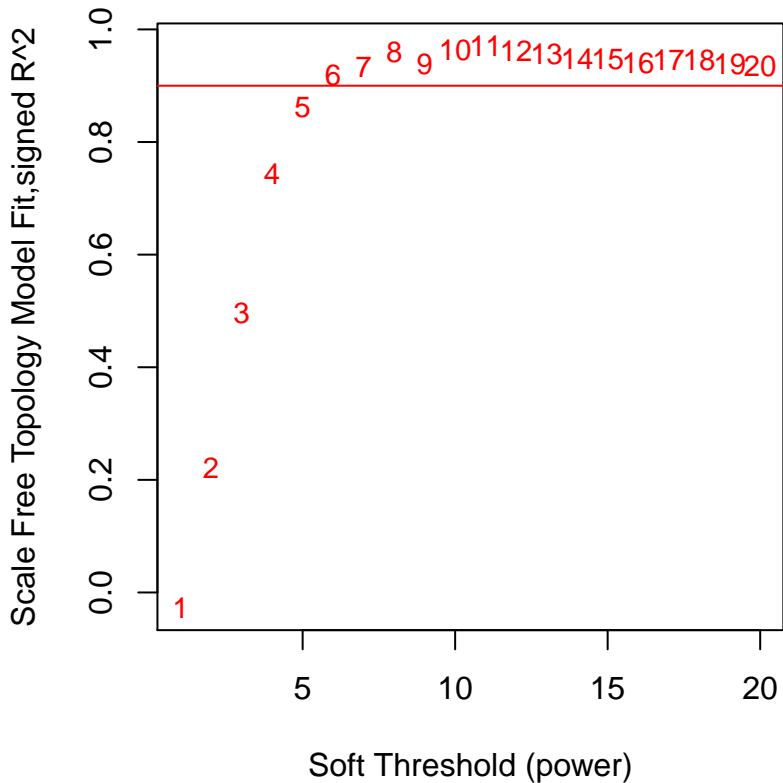

### Mean connectivity

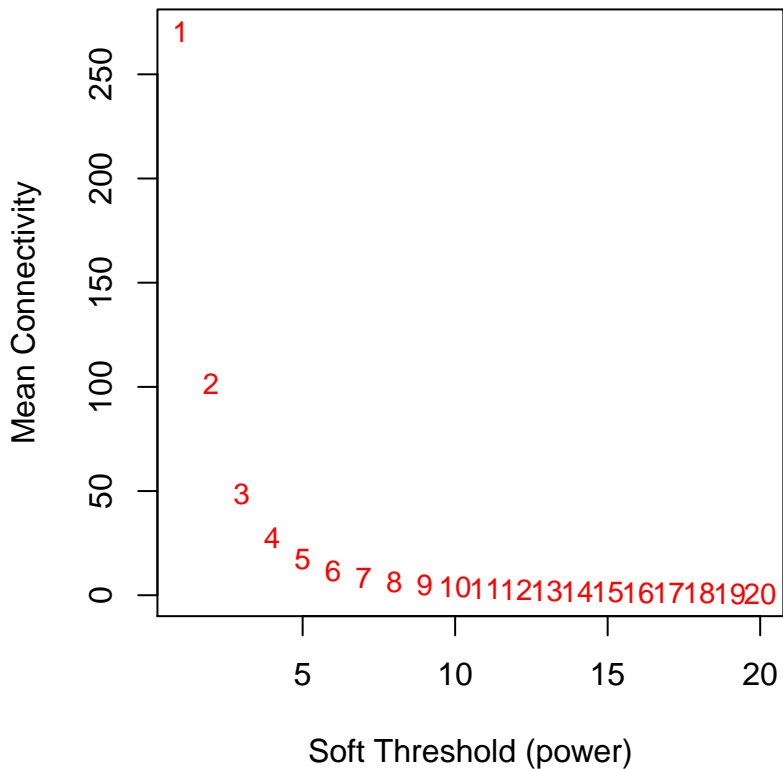

Supplement: Supplementary file 1 [file biomolecules-12-01556-s001.zip › Supplementary Figure S3.pdf]

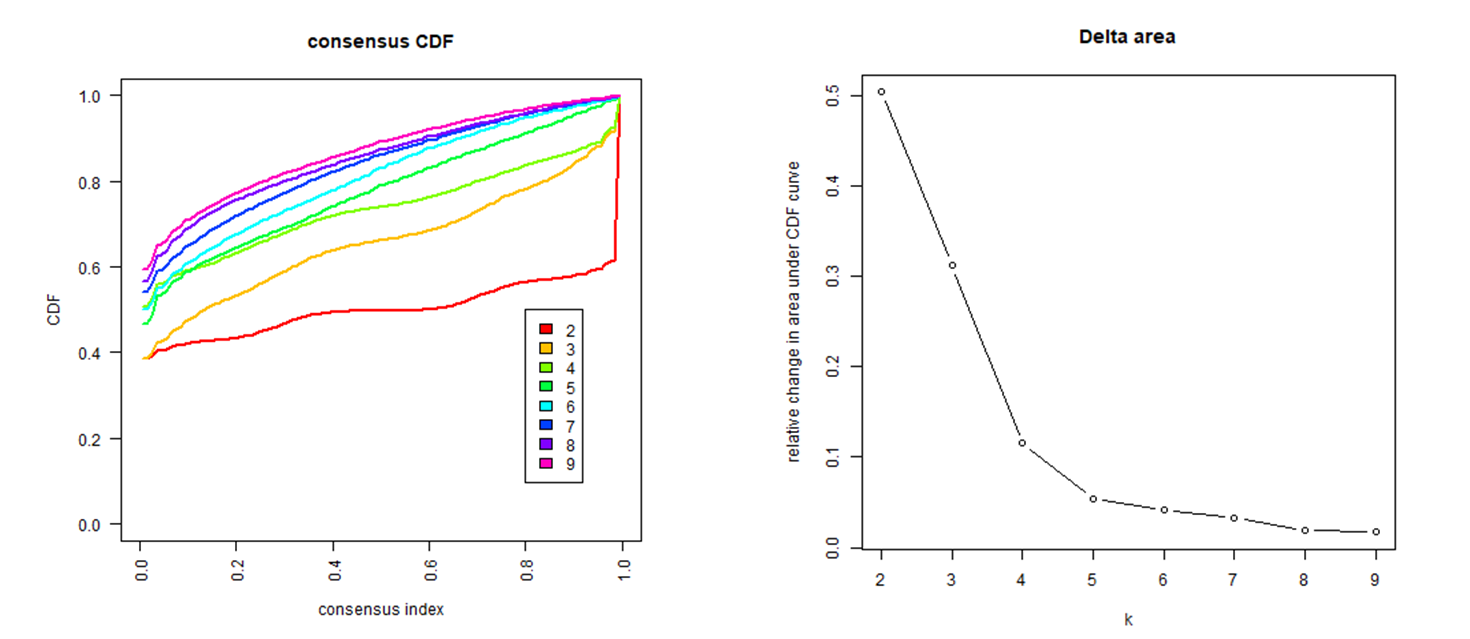

Supplement: Supplementary file 1 [file biomolecules-12-01556-s001.zip › Supplementary Figure S4.tif]

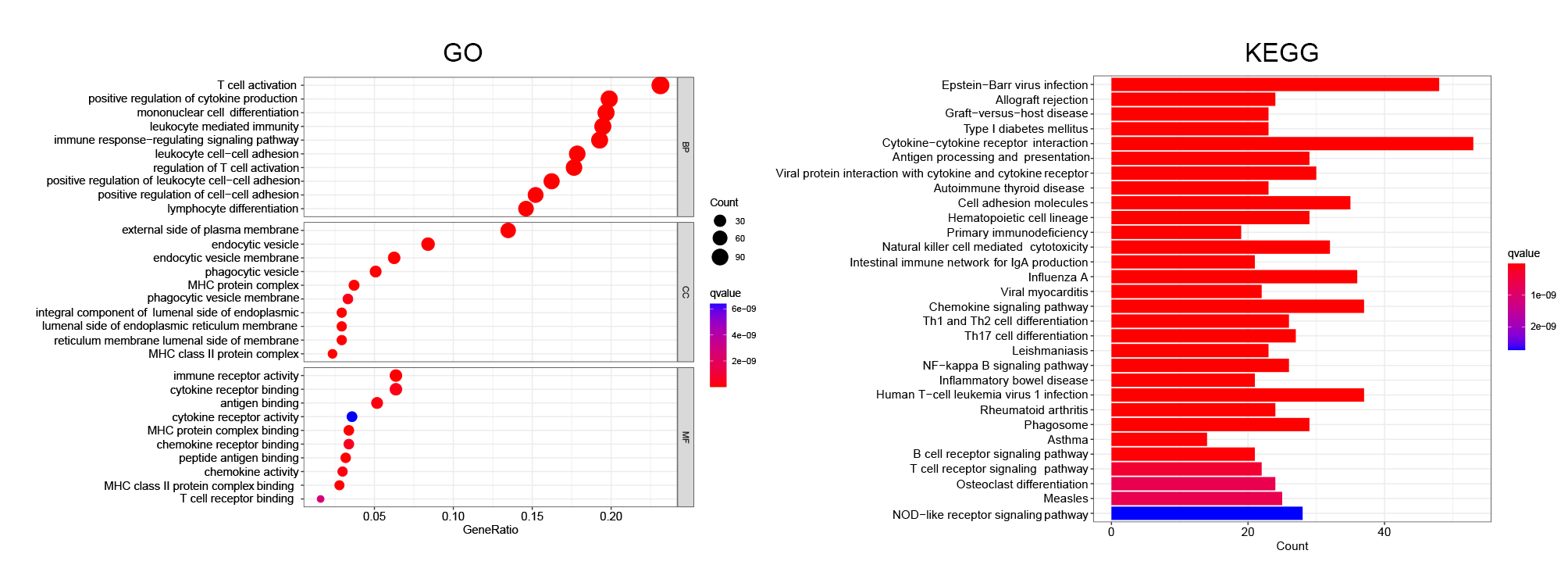

Supplement: Supplementary file 1 [file biomolecules-12-01556-s001.zip › Supplementary Figure S5.tif]

Low-risk High-risk

0.057

Tumor Mutation Burden

20  
15  
10  
5  
0

Low-risk

High-risk

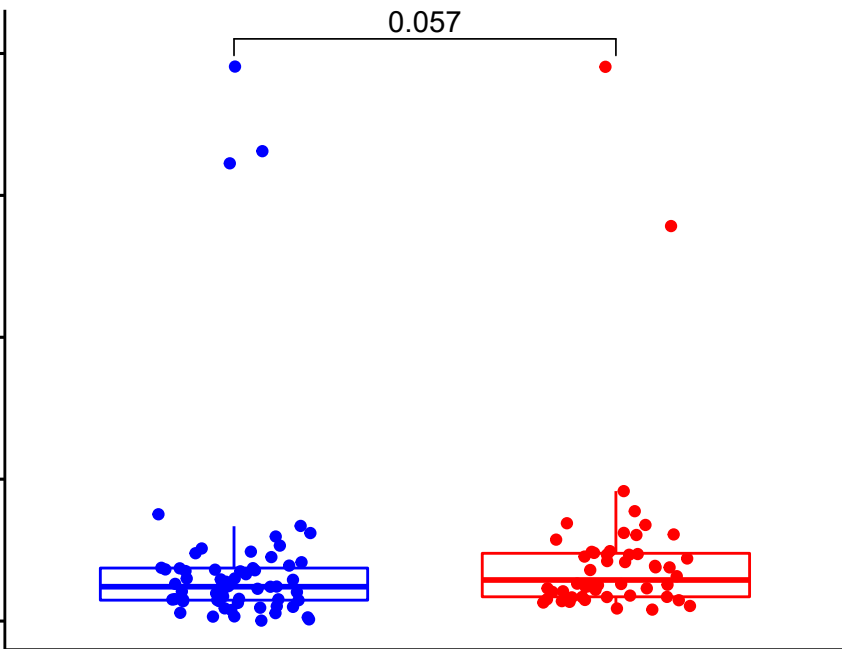

Supplement: Supplementary file 1 [file biomolecules-12-01556-s001.zip › Supplementary Figure S6.pdf]

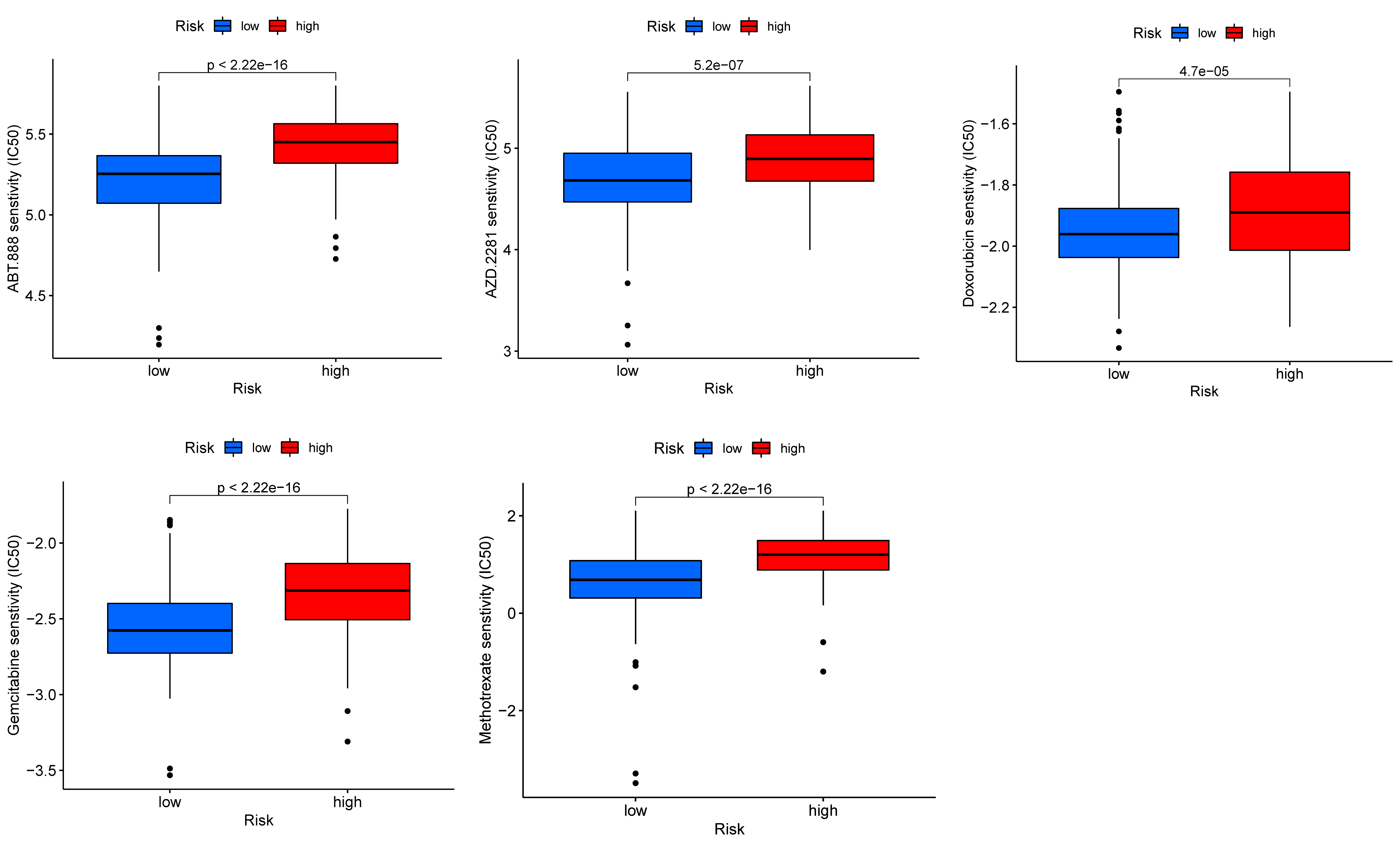

Supplement: Supplementary file 1 [file biomolecules-12-01556-s001.zip › Supplementary Figure S7.tif]
